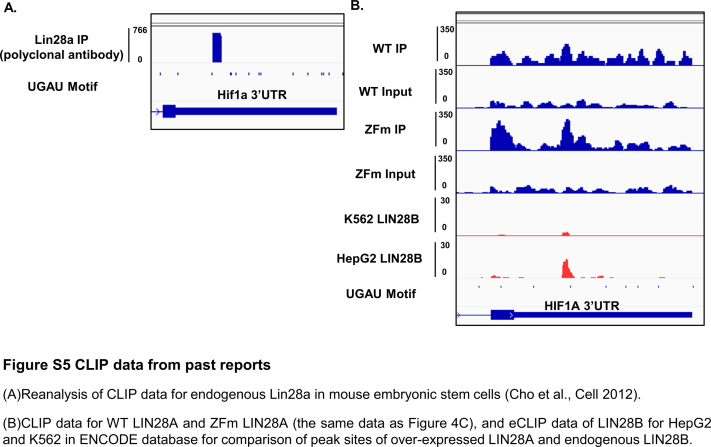# RNA-binding protein LIN28A upregulates transcription factor HIF1α by posttranscriptional regulation *via* direct binding to UGAU motifs

**DOI:** 10.1016/j.jbc.2022.102791

**Published:** 2022-12-09

**Authors:** Hiroto Yamamoto, Yutaro Uchida, Ryota Kurimoto, Tomoki Chiba, Takahide Matsushima, Yoshiaki Ito, Maiko Inotsume, Kohei Miyata, Kenta Watanabe, Masaki Inada, Naoki Goshima, Tokujiro Uchida, Hiroshi Asahara

**Affiliations:** 1 Department of Systems BioMedicine, Tokyo Medical and Dental University (TMDU), Tokyo, Japan; 2 Department of Anesthesiology, Tokyo Medical and Dental University (TMDU), Tokyo, Japan; 3 Department Obstetrics and Gynecology, Faculty of Medicine, Fukuoka University, Fukuoka, Japan; 4 Department of Biotechnology and Life Science, Tokyo University of Agriculture and Technology, Tokyo, Japan; 5 Biomedicinal Information Research Center, The National Institute of Advanced Industrial Science and Technology, Tokyo, Japan; 6 Department of Human Science, Faculty of Human Science, Musashino University, Tokyo, Japan; 7 Department of Molecular and Experimental Medicine, The Scripps Research Institute, San Diego, California, USA

**Keywords:** cancer, cell-based screening, CLIP, hypoxia-inducible factor, LIN28A, RNA, RNA-binding protein, RNA-protein interaction, cDNA, complementary DNA, CSDm, cold shock domain, DMEM, Dulbecco’s modified Eagle’s medium, eCLIP, enhanced crosslinking immunoprecipitation, HIF, hypoxia-inducible factor, RBP, RNA-binding protein, RIPA, rapid immunoprecipitation, qPCR, quantitative PCR, ZFm, zinc finger mutant

## Abstract

Hypoxia-inducible factor 1α (HIF1α) is a transcription factor that regulates angiogenesis under hypoxic conditions. To investigate the posttranscriptional regulatory mechanism of HIF1α, we performed a cell-based screening to reveal potential *cis*-elements and the regulatory RNA-binding proteins that act as *trans*-factors. We found that LIN28A promoted HIF1α protein expression independently of the downregulation of microRNA let-7, which is also directly mediated by LIN28A. Transcriptome analysis and evaluation of RNA stability using RNA-seq and SLAM-seq analyses, respectively, revealed that LIN28A upregulates HIF1A expression *via* mRNA stabilization. To investigate the physical association of LIN28A with *HIF1A* mRNA, we performed enhanced crosslinking immunoprecipitation in 293FT cells and integrally analyzed the transcriptome. We observed that LIN28A associates with *HIF1A* mRNA *via* its *cis*-element motif “UGAU”. The “UGAU” motifs are recognized by the cold shock domain of LIN28A, and the introduction of a loss-of-function mutation to the cold shock domain diminished the upregulatory activities performed by LIN28A. Finally, the microvessel density assay showed that the expression of LIN28A promoted angiogenesis *in vivo*. In conclusion, our study elucidated the role of LIN28A in enhancing the HIF1α axis at the posttranscription layer.

Under certain stressful conditions, tumor progression may continue or even be promoted, whereas normal cells and organs are damaged as they fail to maintain homeostasis (1, 2, 3). Under hypoxic conditions, hypoxia-inducible factors (HIFs) play a central role in angiogenesis and glycolysis in tumor survival (1, 4, 5, 6, 7). Among HIFs, HIF1α is the key protein for tumor survival, and its expression is tightly regulated in multiple steps. The phosphoinositide 3-kinase-Akt and PKC pathways are involved in transcriptional regulation (5). The posttranslational regulation of HIF1α by the prolyl hydroxylase domain protein, and the von Hippel-Lindau protein, are dominant and well-characterized mechanisms of HIF1α regulation (8).

Posttranscriptional effects are another critical step in regulating HIF1α. In general, mRNA stability is strictly regulated by RNA-binding proteins (RBPs) and noncoding RNAs (9). Adenylate-uridylate–rich elements in the 3′-UTR of *HIF1A* mRNA act as critical *cis*-elements for posttranscriptional regulation by ZFP36L1 (10). Moreover, the RBPs, HuR and PTB, act cooperatively to regulate the expression of HIF1α posttranscriptionally, but the *cis*-elements for their function have not been identified (11). These findings prompted us to survey other potential regulatory mechanisms for HIF1α expression by studying its *cis*-elements and the corresponding RBPs.

For this, we designed a cell-based screening assay for 1127 RBPs in 293FT cells by cotransfecting a luciferase reporter containing the UTRs of *HIF1A* mRNA. We identified LIN28A as a *trans*-factor that promotes mRNA stabilization *via* the “UGAU” *cis*-elements in the 3′-UTR of *HIF1A* mRNA. Interestingly, upregulation of HIF1α by LIN28A was independent of the let-7 pathway, providing a novel angiogenic pathway driven by LIN28A *via* “UGAU” motifs in the targeted mRNAs.

## Results

### LIN28A upregulates the expression of HIF1α independently of let-7 degradation

**Figure 1:**
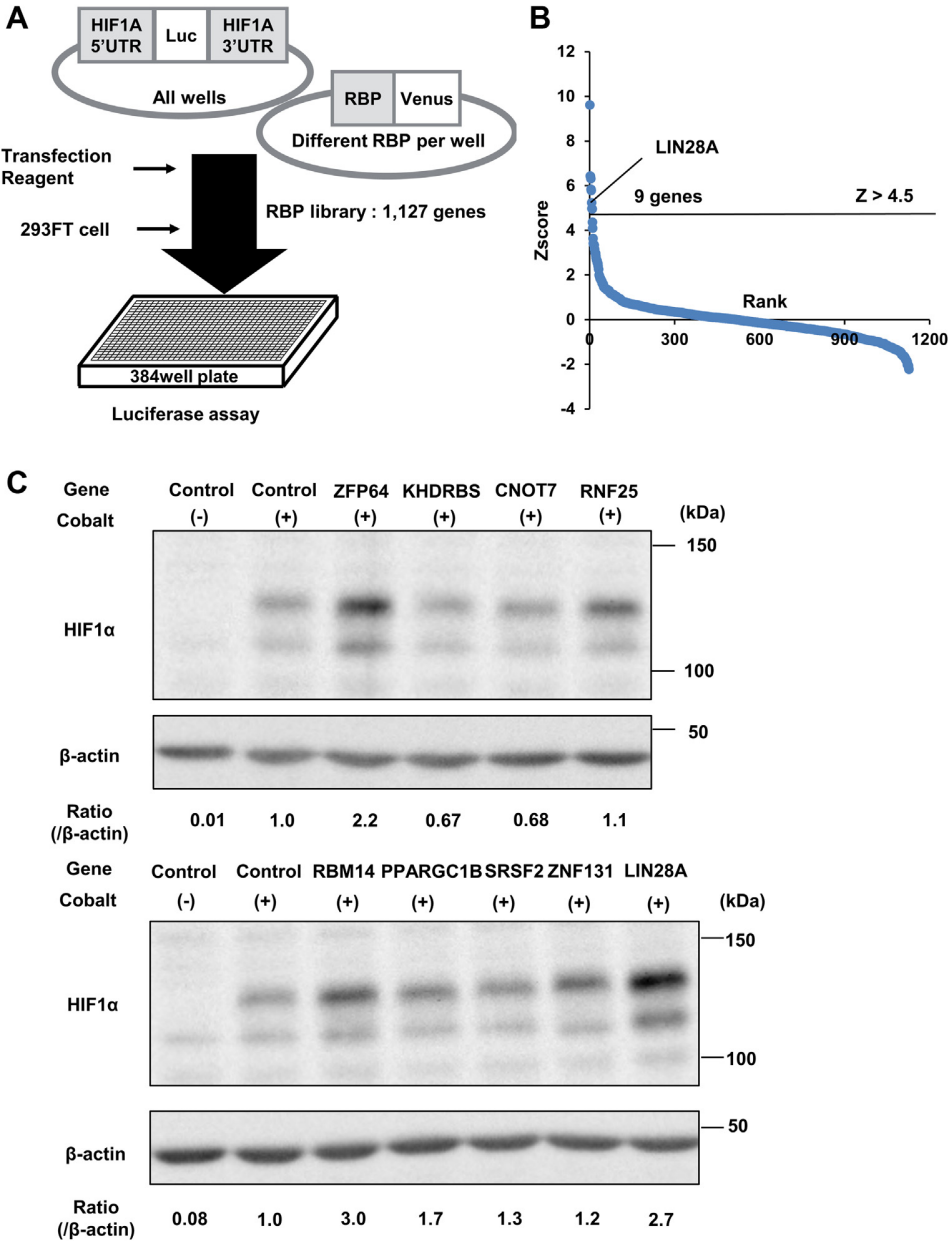
**RNA-binding proteins screening identifies LIN28A as the upregulator of HIF1α.***A*, RNA-binding protein screening by the coexpression of 1127 RNA-binding proteins and the luciferase reporter containing 5′-UTR and 3′-UTR. *B*, ranking graph for Z-scores of the RNA-binding protein screening. *C*, Western blot of the samples expressing candidate RNA-binding proteins in the second screening. HIF, hypoxia-inducible factor.

**Supporting Figure S1:**
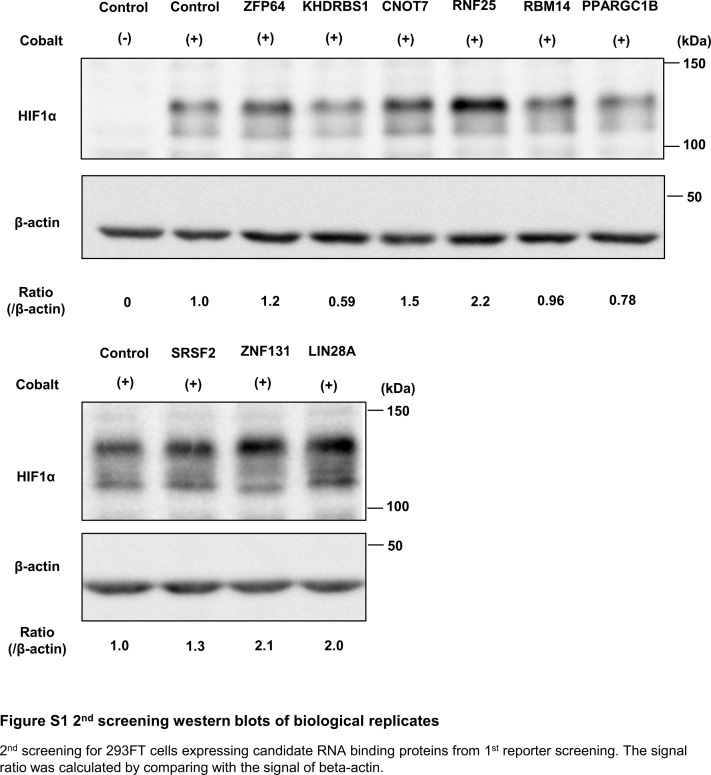


To identify the RBP that regulates the expression of HIF1α posttranscriptionally, we screened RBPs using a cell-based assay system. A luciferase reporter was created containing the 5′- and 3′-UTRs of HIF1A mRNA. We cotransfected this reporter with plasmids expressing 1127 RBPs (Fig. 1*A*). The relative luciferase activity was measured in comparison to the luciferase activity of samples transfected with empty vector, and the Z-scores were calculated from the average and variance of luciferase activity. Nine RBPs with Z-scores >4.5 were identified (Fig. 1*B* and Table S1). Furthermore, to identify the RBP regulating endogenous HIF1α, we performed western blots on samples expressing these nine RBPs as a second screening under the cobalt chloride treatment to prevent the degradation of HIF1α protein. LIN28A was identified as a novel RBP that upregulates the expression of HIF1α (Figs. 1*C* and S1).

**Figure 2:**
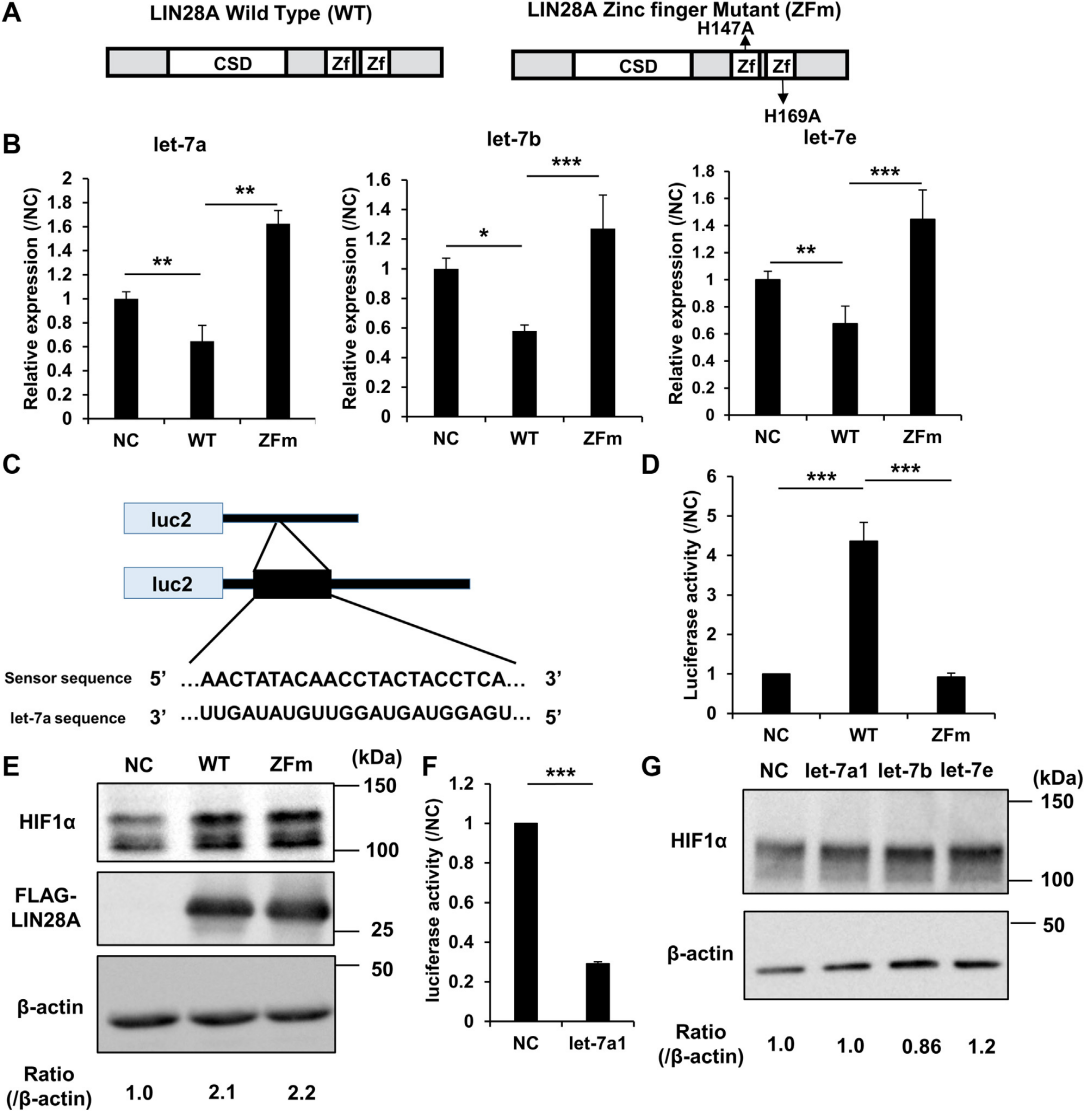
**The upregulation of HIF1α by LIN28A is independent of the degradation of let-7.***A*, schema for the WT and zinc finger mutant (ZFm) LIN28A. LIN28A is composed of a cold shock domain (CSD) and two zinc fingers (Zf). *B*, results of let-7 (let-7a, let-7b, let-7e) TaqMan qPCR for 293FT samples expressing the WT or the ZFm LIN28A and the negative control (NC) with cobalt chloride stimulation. Comparisons between samples were evaluated by Tukey’s test between three groups (N = 3). Data are presented as means ± SD. ∗*p* < 0.05, ∗∗*p* < 0.01, ∗∗∗*p* < 0.005. *C*, schema for the sensor vector of let-7. The antisense sequences for let-7a1 are presented on the sensor vector. *D*, luciferase assay for the let-7 sensor with LIN28A and its mutant. Luciferase activity of the sensor vector was used to examine 293FT samples expressing the negative control (NC), WT, or ZFm LIN28A and let-7 with cobalt chloride stimulation. Samples were evaluated by Tukey’s test between three groups (N = 3). Data are presented as means ± SD. ∗∗∗*p* < 0.005. *E*, Western blot for 293FT cells expressing the NC or WT and ZFm LIN28A with cobalt chloride stimulation. *F*, luciferase assay of the let-7 sensor. Luciferase activity of the let-7 sensor was used to examine 293FT samples expressing the NC or let-7a1 with cobalt chloride stimulation. Samples were evaluated by two-tailed unpaired Student’s *t*-tests (N = 3). Data are represented as mean ± SD. ∗∗∗*p* < 0.005. *G*, Western blot for 293FT cells expressing the negative control (NC) or let-7a1, let-7b, and let-7e. HIF, hypoxia-inducible factor; qPCR, quantitative PCR.

**Supporting Figure S2:**
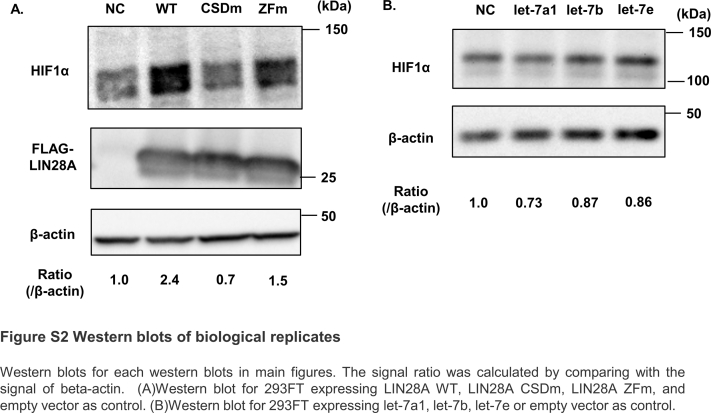


LIN28A is known for its role in suppressing maturation of the tumor-suppressive miRNA, let-7 (12, 13, 14). LIN28A contains cold shock and zinc knuckle domains. His147 and His169 residues of the zinc knuckle domain are essential for the binding and degradation of let-7 (12, 14, 15, 16). Therefore, we created a zinc finger mutant (ZFm) that prevents the degradation of let-7 and verified whether the upregulation of HIF1α was dependent on the degradation of let-7 (Fig. 2*A*). ZFm LIN28A showed a diminished ability to downregulate let-7 compared to WT LIN28A (Fig. 2*B*). Further, we created a sensor vector containing the antisense sequence of let-7a and confirmed that ZFm LIN28A lost the ability to suppress let-7 function, as expected (Fig. 2, *C* and *D*). Interestingly, ZFm LIN28A upregulated HIF1A to the same extent as the WT LIN28A (Figs. 2*E* and S2*A*).

**Supporting Figure S3:**
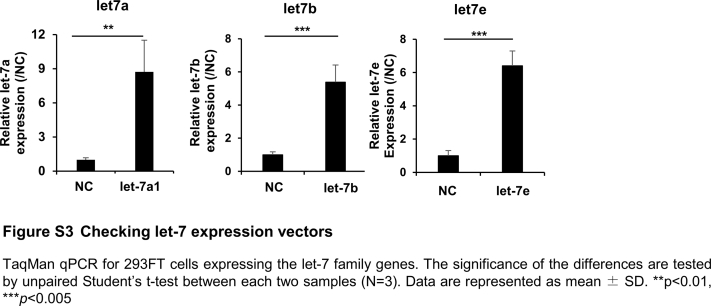


To confirm whether let-7 suppressed HIF1α, we overexpressed the representative let-7 family members, let-7a and let-7e, and a sensor and performed a Western blot for HIF1α (Fig. S3). As expected, let-7 did not suppress the expression of HIF1α, whereas sensor luciferase activity was downregulated by let-7a under the same conditions (Figs. 2, *F* and *G* and S2*B*). These results indicate that LIN28A upregulates the expression of HIF1α independent of let-7 degradation.

### LIN28A upregulates HIF1α by directly binding to the mRNA UGAU motifs with its cold shock domain

**Figure 3:**
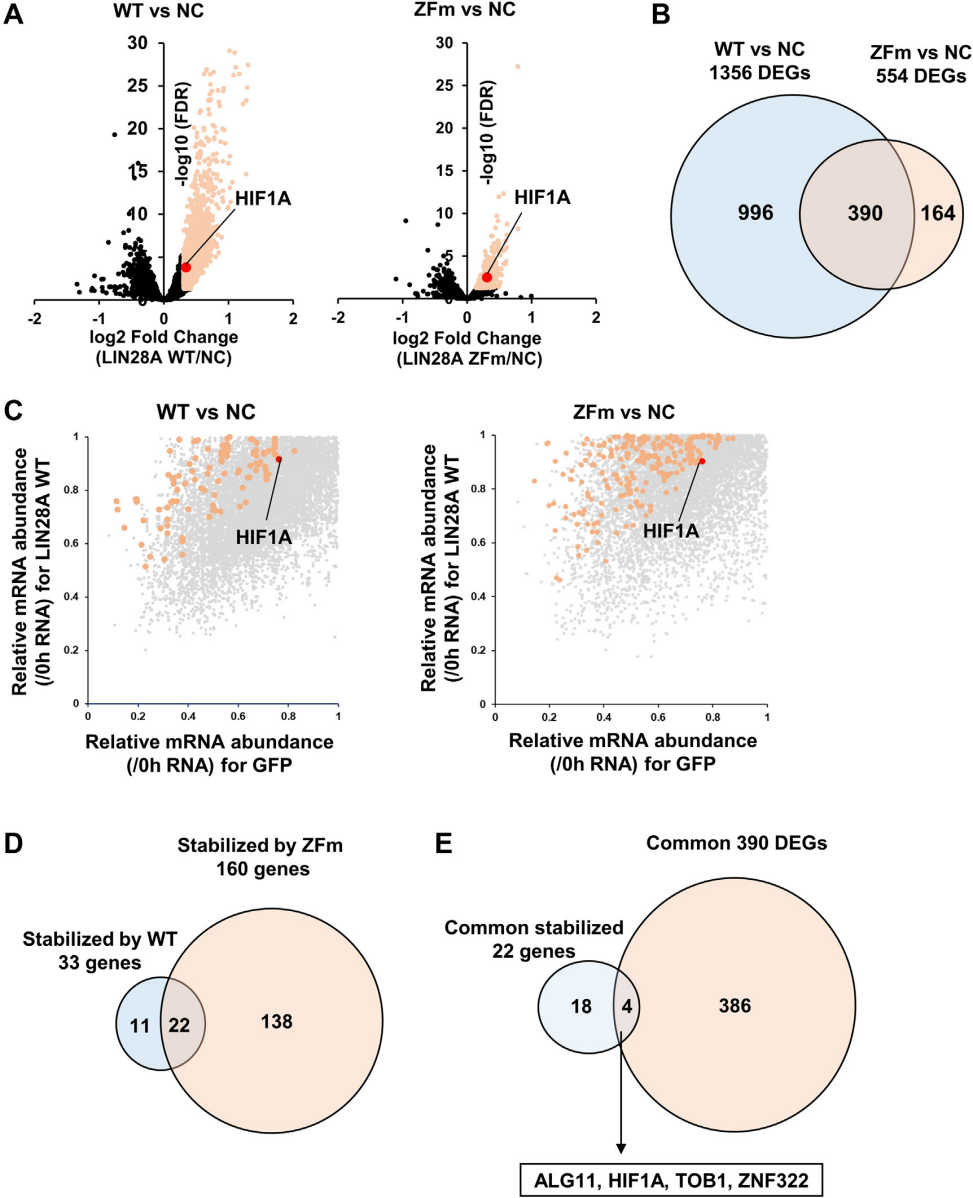
**LIN28A stabilized set of genes including HIF1A independently from let-7 degradation.***A*, volcano plots for RNA-seq of 293FT cells expressing negative control (NC), WT, or zinc finger mutant (ZFm) of LIN28A. *B*, Venn diagram for the upregulated differentially expressed genes (DEGs). *C*, results of SLAM-seq. Distribution plots show the average remaining RNA ratio (compared to T>C conversion ratio at 0 h) in triplicate experiments (N = 3) for each sample (293FT cells expressing negative control [NC], WT, or ZFm version of LIN28A). *Orange plots* show significantly stabilized transcripts. One-way ANOVA with Dunnett’s multiple comparisons post hoc test was performed to statistically analyze the differences between each sample and the negative control. Transcripts with *p* < 0.05 and a remaining RNA ratio >1.1 fold were considered statistically significantly modulated compared to the negative control. *D*, Venn diagram for the common stabilized genes by WT or ZFm expression. *E*, Venn diagram and gene lists for the common stabilized genes measured by SLAM-seq and common DEGs from RNA-seq. HIF, hypoxia-inducible factor.

LIN28A suppresses the maturation of let-7 and regulates translation by binding directly to target mRNAs (17, 18). To determine the genes upregulated by the direct binding of LIN28A, we performed RNA-seq analysis on 293FT cells expressing WT LIN28A or ZFm LIN28A. WT LIN28A upregulated 1356 genes, including *HIF1A*, compared to the negative control, whereas ZFm LIN28A upregulated 554 genes, including *HIF1A* (Fig. 3*A*). In total, 390 genes were upregulated in 293FT cells expressing both WT and ZFm LIN28A (Fig. 3*B* and Table S2). To further validate whether the upregulation of *HIF1A* by LIN28A depends on *HIF1A* mRNA stabilization, we performed SLAM-seq, which uses s4U, a nucleic acid analog of uridine, to quantify RNA stability (19). The results showed that WT and ZFm LIN28A stabilized the mRNA of 33 genes and 160 genes, respectively. Furthermore, 22 genes were stabilized by both WT and ZFm LIN28A expression (Fig. 3, *C* and *D* and Table S3). Among these, four genes (*ALG11*, *HIF1A*, *TOB1*, and *ZNF322*) were upregulated by both WT and ZFm LIN28A (Fig. 3*E*).

**Figure 4:**
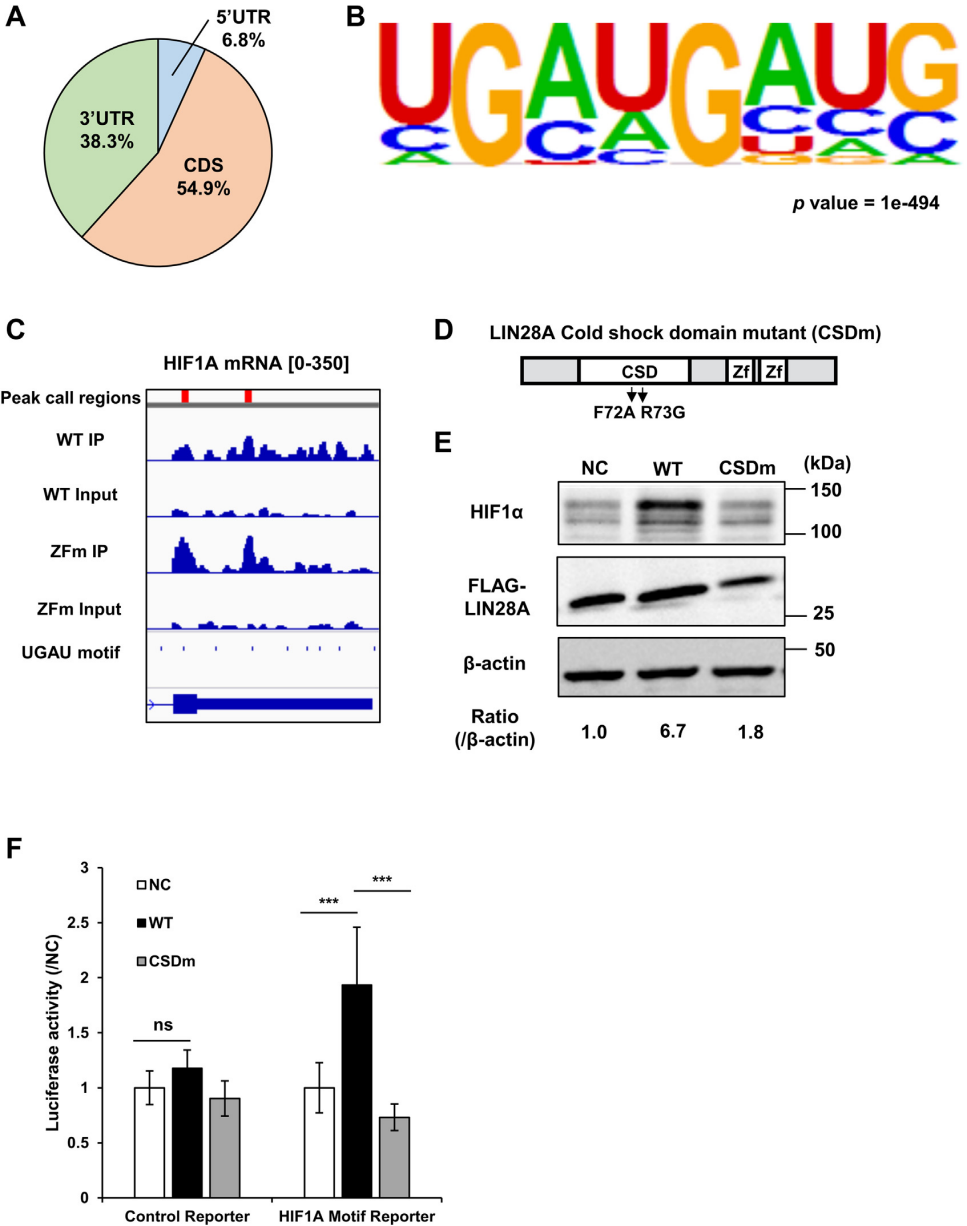
**LIN28A targets UGAU motifs for stabilization of target mRNAs with its cold shock domain.***A*, binding regions of the co-occurring called peaks. *B*, identified motifs from the co-occurring called peaks were analyzed by HOMER. *C*, peak regions from CLIP-seq data for *HIF1A*, *PPP2R5E*, and *YTHDF3* mRNAs. Numbers in *brackets* show the RPKM ranges for each CLIP-seq file. *D*, schema for the cold shock domain mutant (CSDm) of LIN28A. *E*, Western blot analysis of 293FT cells expressing GFP as the negative control (NC) or the WT and CSDm LIN28A with cobalt chloride stimulation. *F*, luciferase assay of reporters containing peak sites with UGAU motifs. Luciferase activity of each reporter was used to examine 293FT samples expressing the NC or WT LIN28A. Samples were evaluated by Tukey’s test between three groups (N = 6). ∗∗∗*p* < 0.005, ns: nonsignificant. HIF, hypoxia-inducible factor.

**Supporting Figure S4:**
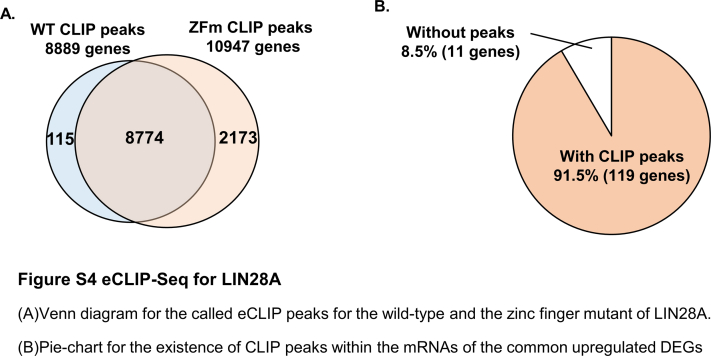


Next, we performed enhanced crosslinking immunoprecipitation (eCLIP)-seq on 293FT cells expressing WT LIN28A or ZFm LIN28A to identify the transcriptome-wide–binding sites and binding motif elements of LIN28A. The results showed 8774 coding genes with co-occurring CLIP peaks for WT LIN28A and ZFm LIN28A (Fig. S4). Analysis of the CLIP peaks showed that LIN28A dominantly binds to the coding sequence and the 3′-UTRs of mRNAs (Fig. 4*A*). Furthermore, a motif analysis of the co-occurring CLIP peaks revealed that LIN28A was bound to the UGAU motif (Fig. 4*B*). Consistent with the transcriptome-wide analysis, LIN28A bound to the UGAU motif in the 3′-UTR of *HIF1A* mRNA (Fig. 4*C*). The identified UGAU motifs were previously found to be bound by the cold shock domain (CSDm) of LIN28A (20). Therefore, by changing the Phe72 and Arg73 residues in CSDm, we created a loss-of-function mutant as described previously (Fig. 4*D*) (21). As a result, the introduction of a mutation in the cold shock domain inhibited HIF1A upregulation (Figs. 4*E* and S2*A*). Besides, to evaluate the effect of the binding sites on the effector activity of LIN28A, we performed a luciferase reporter assay for reporters containing UGAU motifs–associated *HIF1A*-binding sites. The results of the reporter assay showed that WT LIN28A upregulates reporter activity, while CSDm LIN28A loses the ability to upregulate the reporter activity (Fig. 4*F*).

### LIN28A promotes angiogenesis *in vivo*

**Figure 5:**
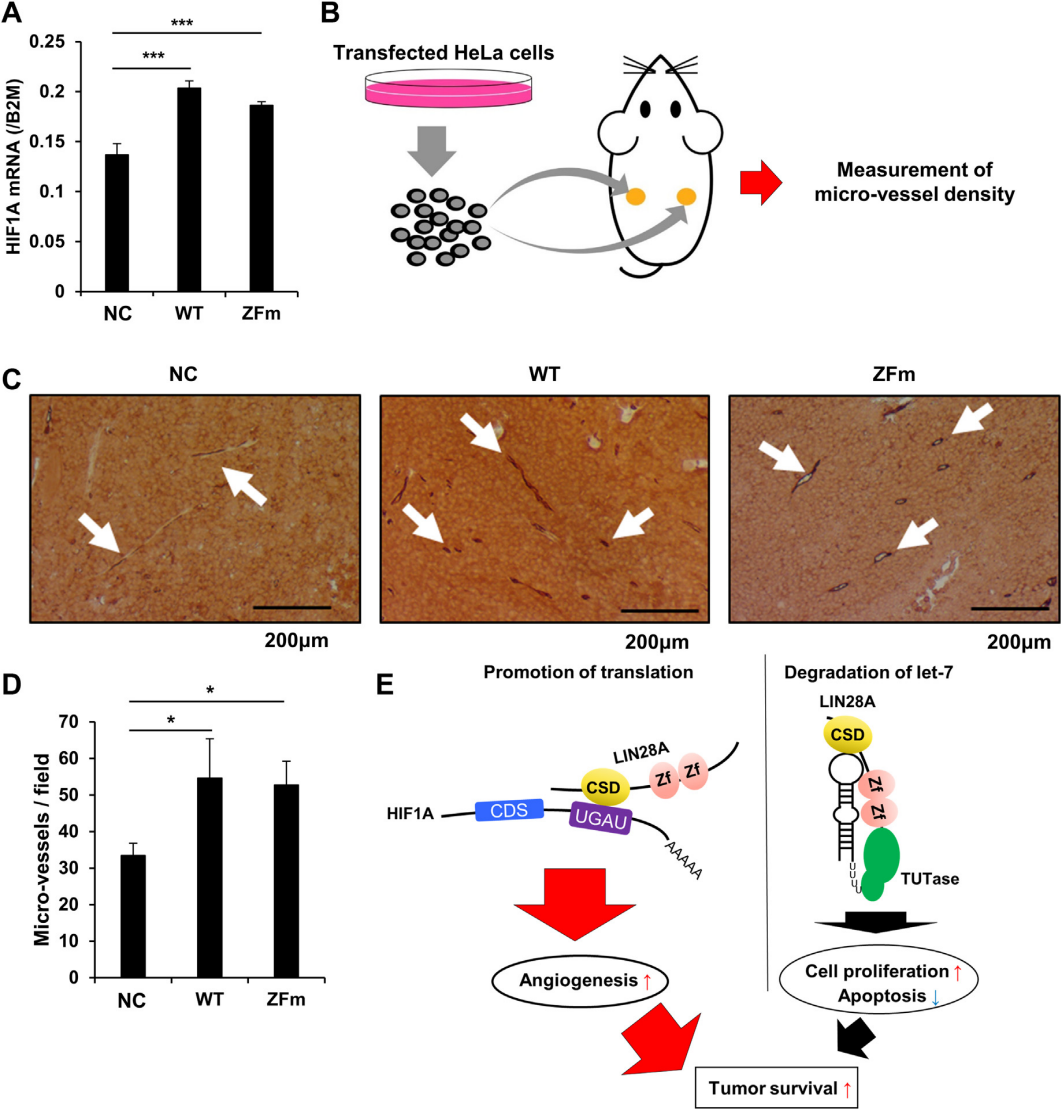
**LIN28A promotes angiogenesis *in vivo*.***A*, qPCR of *HIF1A* in HeLa cells expressing the WT or zinc finger mutant (ZFm) LIN28A or GFP as a negative control (NC). Differences between samples were evaluated by Tukey’s test between three groups (N = 3). Data are presented as means ± SD. ∗∗∗*p* < 0.005. *B*, schema for the *in vivo* xenograft assay. HeLa cells (1 × 10^7^) infected with lentivirus expressing GFP or WT LIN28A or ZFm containing FLAG-peptide were transplanted subcutaneously using 100 μl of PBS in 6-week-old female nude mice. The transplanted tumor was grown until it reached 3 cm × 3 cm and the microvessel density was measured. *C*, pathological images of xenografted HeLa cells expressing the WT or ZFm LIN28A or GFP as a NC. The pathological slides of xenografted tumors were stained with murine Cd34 to examine the density of microvessels. *D*, microvessel density was calculated for the xenografted samples. The number of microvessels per high-power field (×400) was calculated for three fields of view. Differences between negative control were evaluated by Dunnett’s test (N = 3). Data are presented as means ± SD. ∗*p* < 0.05. *E*, our proposed model of the novel LIN28A–HIF1A axis. LIN28A can upregulate HIF1A independently of the degradation of let-7 by direct binding to *cis*-regulatory elements of target mRNAs. HIF, hypoxia-inducible factor; qPCR, quantitative PCR.

To determine the significance of HIF1α upregulation by LIN28A *in vivo*, we used a microvessel assay to examine the promotion of angiogenesis by HIF1α. WT LIN28A and ZFm LIN28A were overexpressed in HeLa cells to confirm the upregulation of HIF1α (Fig. 5*A*). These cells were then transplanted subcutaneously into nude mice and changes in angiogenesis were detected (Fig. 5*B*). By counting the number of microvessels per high-power field, we found that tumors expressing WT LIN28A and ZFm LIN28A showed significantly enhanced angiogenesis (Fig. 5, *C* and *D*). Here, we suggest the novel roles of LIN28A in upregulating genes important for tumor survival by direct binding to *cis*-elements of their mRNAs, and this upregulation is independent of the degradation of let-7 (Fig. 5*E*).

## Discussion

In this study, we performed cell-based screening to identify RBPs regulating HIF1α. We found that LIN28A regulated the expression of HIF1α. LIN28A is a well-known RBP that degrades the tumor-suppressing miRNA, let-7, by inducing its oligo-uridylation by TUTases (12, 16). However, our analysis suggested that LIN28A regulates HIF1α by recognition of UGAU motifs in 3′-UTR of mRNA with its CSDm and the stabilization of mRNAs and independently of the degradation of let-7. Besides, our *in vivo* experiment indicated that the expression of LIN28A contributed to tumor angiogenesis.

LIN28A is a crucial RBP identified as a progenitor self-renewal promoting factor in the development of *Caenorhabditis elegans* larvae (22). The most important role of LIN28A is to suppress maturation of the tumor-suppressive miRNA, let-7. LIN28A binds to let-7 on the GGAG site with its zinc knuckle domain, resulting in the recruitment of TUTases. TUTases polyuridylate and rapidly degrade let-7 (12, 15, 16). The LIN28A–let-7 axis has been reported to contribute to homeostasis in multiple aspects (13, 18, 23, 24). Moreover, LIN28A also has some downregulatory roles independent of let-7 degradation by directly binding to target mRNAs (17). Our motif analysis and binding sites analysis from eCLIP-seq data revealed that LIN28A bound to transcripts containing UGAU motifs in 3′-UTRs of target mRNAs. A similar CLIP approach has been utilized by Hafner *et al.* (25) for analyses of HEK293 cells overexpressing LIN28A. Based on GSE44616 (GEO datasets), the peak call data for LIN28A showed a binding peak in the same UGAU site in the 3′-UTR of *HIF1A* mRNA, identical to our eCLIP data. The reanalysis of CLIP-seq data for endogenous Lin28a in murine embryonic stem cells obtained by Cho *et al.* (17) showed that endogenous Lin28a binds to a similar UGAU site in the 3′-UTR of *Hif1a* mRNA (Fig. S5*A*). Besides, we referred to the eCLIP data for endogenous LIN28B, deposited in the ENCODE database (26). LIN28B is an isoform of LIN28A sharing domains with high homology. The available eCLIP data for endogenous LIN28B in K562 and HepG2 cells showed a similar endogenous LIN28B-binding peak to our identified WT and ZFm LIN28A peaks near UGAU motifs in the 3′-UTR of *HIF1A* mRNA (Fig. S5*B*). These reanalysis support the reliability of our finding that UGAU motifs in *HIF1A* mRNA are physically targeted by LIN28A.

LIN28A is also known for its roles in epithelial to mesenchymal transition and contributes to tumor malignancies in various cancer types (27, 28, 29, 30, 31, 32, 33, 34). Our transcriptome and SLAM-seq analyses revealed that LIN28A upregulated and stabilized *HIF1A* mRNA. It is also possible that LIN28A upregulates the expression of HIF1A by promoting translation activity, although we did not verify this possibility in this study. Further studies are needed to clarify the role of LIN28A in translational activity for the upregulation of transcripts containing UGAU.

Moreover, we revealed that the CSDm of LIN28A is essential for recognizing UGAU motifs in target mRNAs. A structural analysis of LIN28A has shown that the CSDm of LIN28A recognizes the “NGAY” motif (35). The recognition of “UGAU” motif *via* the CSDm of LIN28A was shown to be essential for the functional interaction with let-7 by CLIP analyses and biochemical approaches (20). These past reports support our finding that LIN28A recognizes UGAU motifs of mRNAs. Therefore, the function of LIN28A in mRNAs *via* its CSDm can be inferred to upregulate a set of genes involved in angiogenesis. We also observed that LIN28A promotes angiogenesis *in vivo* in let-7–independent manner and infer that this novel LIN28A–HIF1A axis contributes to tumor survival in the body.

As a limitation of this study, we could not perform experiments on endogenous LIN28A because of the difficulties in obtaining cell lines expressing high level of LIN28A. However, our study shows the potential roles of LIN28A in tumor angiogenesis, which was consistent with the previous findings highlighting the importance of LIN28A expression in tumorigenesis pathologically. Future research should focus on delineating the role of endogenous LIN28A in angiogenesis.

In conclusion, we identified LIN28A as an RBP that upregulates the expression of HIF1α in 293FT cells independent of the degradation of let-7. LIN28A binds directly to and upregulates HIF1A mRNAs with the UGAU motifs. For the regulatory function by LIN28A, the recognition of UGAU motifs in target mRNAs *via* CSDm is essential. The let-7–independent upregulation of HIF1α is also significant for the promotion of angiogenesis *in vivo*.

## Experimental procedures

The brief experimental procedures are shown here. The detailed materials and methods are also described in Supporting Information S1.

### Animal experiments

All the animal experiments in this study were conducted according to the Guidelines for Proper Conduct of Animal Experiments (Science Council of Japan) and approved by the Center for Experimental Animals of Tokyo Medical and Dental University (Approval No. A2020-091A).

### Plasmid construction

Detail information for constructs of plasmids is described in Supporting Information S1 and S2.

### RBP screening

Twenty nanograms of RBP expression vectors, 10 ng of luciferase reporter containing UTRs of the *HIF1A* gene upstream and downstream of the luciferase gene, and 5 ng of pRL-SV40 vector are transfected with 0.15 μl of Fugene HD Transfection Reagent (Promega), 5 μl Opti-MEM (Thermo Fisher Scientific), and 2 × 10^3^ 293FT cells in 40 μl of Dulbecco’s modified Eagle’s medium (DMEM) with 10% fetal bovine serum and 1% penicillin-streptomycin in 384-well plates. Forty-eight hours later, the luciferase activity was measured using ARVO X3 (PerkinElmer) with the Dual-Glo Luciferase Assay System (Promega). As a control, we used a vector expressing Venus (pcDNA3.1-Venus) and calculated the relative luciferase activities by comparing the luciferase activities of each RBP and negative controls. The duplicate experiments (N = 2) were performed for each RBP. The average and variance of the relative luciferase activity were calculated, and the Z-score for each RBP was determined.

### Second screening

We plated 6 × 10^5^ 293FT cells in 6-well plates with 3 ml of DMEM. After 24 h, 2 μg of vectors expressing RBPs are transfected with 7 μl of PEI and 100 μl of Opti-MEM. After 24 h, CoCl_2_ was added at a final concentration of 250 μM, and the cells were incubated for 24 h. We precipitated the proteins with rapid immunoprecipitation (RIPA) buffer (50 mM Tris–HCl, 150 mM NaCl, 1% NP-40, 0.5% sodium deoxycholate, 0.1% SDS) and performed Western blot following the given protocol. The experiment was performed for two times and the signal ratio compared with beta-actin was calculated.

### Western blotting

Proteins were separated by SDS-PAGE, followed by semidry transfer to a PVDF membrane. Membranes were blocked with Blocking-One (Nacalai Tesque) for 60 min and made to react with primary antibodies against HIF1α (610958, BD Biosciences), LIN28A(H-44, Santa Cruz Biotechnology), FLAG (F3165, Sigma-Aldrich), and ACTB (AC-74, Sigma-Aldrich) at 4 °C overnight. After washing with phosphate buffered saline with Tween 20, the membranes were made to react with ECL mouse IgG HRP-conjugated whole antibody (GE Healthcare). After washing with phosphate buffered saline with Tween 20, the blot was developed using Pierce ECL Western Blotting Substrate (Thermo Fisher Scientific) and detected with LAS 4000 (GE Healthcare).

### Overexpression of each mutant of LIN28A

For transfection experiments, 9 × 10^5^ 293FT cells were plated on a 6-well plate. After 24 h, 2 μg of vectors were transfected using 7 μg of PEI. CoCl_2_ was added after 24 h with a final concentration of 250 nM. After 24 h, we collected the proteins using RIPA buffer and performed a Western blot or collected RNA using TRIzol and analyzed by qRT-PCR as mentioned below. For experiments with Tet-on cells, we created 293FT cells expressing WT LIN28A and CSDm LIN28A with Tet-on system. Briefly, 293FT cells were infected with lentivirus expressing GFP, WT, CSDm, or ZFm with FLAG-peptide in Tet-on system. After 24 h, cells were selected using 1 μg/ml puromycin for 2 to 3 days. The created Tet-on–expressing cells were treated with 100 ng/ml of doxycycline (Wako) and 250 nM of CoCl2. After 24 h, we collected the proteins using RIPA buffer and performed a Western blot as mentioned above. The protein expression changes for each mutant was calculated for total two times (N = 2) for each LIN28A mutants and the signal ratio compared with beta-actin was calculated. For *in vivo* experiments, HeLa cells were infected with lentivirus expressing GFP or ZFm LIN28A with FLAG-peptide. After 24 h, cells were selected using 1 μg/ml puromycin for 2 to 3 days. After selection, CoCl_2_ was added to a final concentration of 250 nM and incubated for 24 h. RNA was isolated using TRIzol and analyzed by quantitative PCR (qPCR) as mentioned above. The qPCR experiment was performed for three independent samples.

### Quantitative PCR

For the evaluation of mRNA expression changes, extracted RNA was transcribed using Prime Script (Takara Bio), Super Script III (Thermo Fisher Scientific), or ReverTra Ace (Toyobo) with oligo dT, random primers, and dNTPs. We performed qPCR using Thunderbird SYBR qPCR mix (Toyobo). Complementary DNA (cDNA) was subjected to qPCR using the following primers- *B2M*, 5′- ACTCTCTCTTTCTGGCCTGG-3′ (forward) and 5′-CGTGAGTAACCTGAATCTTTGG -3′ (reverse); *HIF1A* 5′-TGAGCTTGCTCATCAGTTGC-3′ (forward) and 5′-CCATAACAAAACCATCCAAGGC -3′ (reverse). To evaluate miRNA expression, the extracted RNA was reverse-transcribed with TaqMan MicroRNA Reverse Transcription Kit (Thermo Fisher Scientific) and quantitative RT-PCR was run using THUNDERBIRD Probe qPCR Mix (Toyobo). The results are calculated by normalizing to RNU6B expression in each sample. The probes are RNU6B:001093 mature, let-7a:000377, mature let-7b: 000378, mature let-7e:002406 (Thermo Fisher Scientific). The qPCR experiment was performed for three independent samples.

### Let-7 overexpression

For the evaluation of protein expression changes, 4.5 × 10^5^ 293FT cells were plated in 6-well plates. After 24 h, 0.72 μg of the let-7–overexpressing vectors were transfected using PEI (2.52 μl) and 200 μl of Opti-MEM. After 24 h incubation, CoCl_2_ was added at a final concentration of 250 nM, and the plates were incubated for 24 h. We collected the proteins with RIPA buffer and performed a Western blot using the protocols described above. The experiment was performed for two times and the signal ratio compared with beta-actin was calculated. For sensor assay, 6 × 10^4^ 293FT cells were plated in 24-well plates. After 24 h, 210 ng of vectors expressing NC, WT LIN28A, or ZFm LIN28A; 60 ng of vectors expressing the let-7 sensor; and 60 ng of vectors expressing Renilla with SV40 promoter were transfected using 1.89 μl of PEI and 200 μl of Opti-MEM. After incubating for 24 h, CoCl_2_ was added at a final concentration of 250 nM. After 24 h, 180 μl of firefly reagents were added, and the plates were incubated in the dark for 10 min. The luminescence was measured using ARVO X3 (PerkinElmer). We added the same amount of Renilla reagent again and after incubating the plates for 10 min in the dark and measured the luminescence using an ARVO X3 (PerkinElmer). The experiment was performed for three independent samples.

### RNA-seq

We plated 2 × 10^5^ 293FT cells in 35-mm dishes. After 24 h, lentiviruses expressing LIN28A or ZFm were transfected. After 48 h, RNA was collected using Trizol reagent, and 500 ng of the total RNA was used for subsequent preparation. Duplicate experiments (N = 2) were performed for each sample. RNA-seq libraries were prepared using the rRNA-depletion kit (E6310, New England Biolabs Japan) and a directional library synthesis kit (E6310, New England Biolabs Japan). The RNA libraries were sequenced using the NextSeq 500 High-output kit v2 for 2 × 36 base reads. FASTQ files were trimmed with Trim Galore (https://www.bioinformatics.babraham.ac.uk/projects/trim_galore/), mapped with STAR (36), and quantified using RSEM. Differentially expressed genes were standardized and detected using iDEP.91 (37). We treated the genes with expression fold change >0.3 and FDR <0.01 compared with NC as upregulated differentially expressed genes.

### SLAM-seq

s4U (Sigma-Aldrich) in the final concentration of 150 nM was added in DMEM with 10% fetal bovine serum/1% Penicillin-Streptomycin and incubated for 24 h. Following incubation, plates were washed with PBS for two times. Next, media without s4U was added and cells were collected in the time point of 0 h and 4 h. Triplicate samples were collected for each time point. RNA was extracted using TRIzol reagent. The alkylation reaction was performed under standard conditions (50% dimethyl sulfoxide, 10 mM iodoacetamide, 50 mM sodium phosphate pH 8) for 15 min at 50 °C. The reaction was terminated by adding 2 nM DTT. RNA was purified using an RNA Extraction Kit (Zymo Research). An NGS library of the purified RNA was prepared using a Quant-Seq Library Preparation Kit (Lexogen). The prepared library was sequenced using NextSeq 1000 (Illumina). The “SLAM-DUNK” (https://t-neumann.github.io/slamdunk/) software was used for SLAM-seq analysis. Sequence data were aligned to the GRCh38 genome obtained from the Ensembl genome database. Before alignment, adapters of the sequence data were trimmed using Cut Adapt. The trimmed sequence data were then aligned to the GRCh38 genome following the manufacturer's protocol. The T>C conversion rates across different time points were calculated. The remaining RNA ratio (T>C conversion ratio of samples at 4 h) for each sample was calculated by comparing it with the T>C conversion ratio of samples at 0 h. One-way ANOVA with Dunnett’s multiple comparisons post hoc test was performed to statistically analyze the differences between each sample and the negative control (N = 3). Transcripts with *p* < 0.05 and a remaining RNA ratio >1.1 fold were considered statistically significantly modulated compared to the negative control.

### eCLIP

293FT cells expressing WT LIN28A or ZFm LIN28A were UV-crosslinked (254 nm and 300 mJ/cm^2^) using an UV crosslinker (UVP). The proteins were precipitated in lysis buffer (50 mM Tris–HCl (pH 7.4), 100 mM NaCl, 1% NP-40 (Igepal CA630), 0.1% SDS, 0.5% sodium deoxycholate, and protease inhibitor (1:100)) on ice for 15 min. The sample was sonicated using a bioruptor (Cosmo Bio) for 5 min at 4 °C. Next, the sample was incubated with 10 μl RNase I (1:100; Thermo Fisher Scientific) and 2 μl of Turbo DNase (Thermo Fisher Scientific) at 37 °C for 5 min on a thermomixer. The sample was then treated with 11 μl murine RNase inhibitor for 15 min. Immunoprecipitation was performed using the 1:1000 of mouse anti-FLAG antibody (FLA1, MBL), coupled to Dynabeads Protein G (Thermo Fisher Scientific), and incubated at 4 °C for 3 h. RNA–protein complexes were washed with wash buffer (20 mM Tris–HCl [pH 7.4], 10 mM MgCl2, 0.2% Tween-20), high-salt wash buffer (50 mM Tris–HCl [pH 7.4], 1 M NaCl, 1 mM ethylenediaminetetraacetic acid, 1% NP-40, 0.1% SDS, and 0.5% sodium deoxycholate), and Fast AP Buffer (10 mM Tris–HCl (pH 7.4), 5 mM MgCl2, 100 mM KCl, and 0.02% Triton X-100). The bound RNA was incubated with fast alkaline phosphatase (Thermo Fisher Scientific) for 30 min and T4 polynucleotide kinase (NEB) for 45 min. Next, the coupled beads were washed with wash buffer and ligase buffer (50 mM Tris–HCl (pH 7.5), 10 mM MgCl2). The bound RNA was ligated with a 3′-RNA linker for 3 h using RNA ligase high-concentration (NEB) and RNA adapters (AGAUCGAAGAGCGUCGUGUAG). The RNA–protein complex was extracted using NuPAGE sample buffer (Invitrogen) and subjected to SDS-PAGE and were transferred to a nitrocellulose membrane (0.2 μm). The region with the target protein on the blot was cut, and the RNA was extracted using Proteinase K (NEB), acid phenol/chloroform/isoamyl alcohol (Nippon Gene), and Quick-RNA Miniprep kit (Zymo Research). The purified RNA was subjected to reverse transcription using TGIRT-III enzyme (Ingex). The cDNA was incubated with ExoSAP-IT (Thermo Fisher Scientific). The 5′-end of cDNA was ligated with a rand3Tr3 adapter (ACACGACGCTCTTCCGA) using RNA ligase high-concentration (NEB) overnight at room temperature. The adapter-ligated cDNA was PCR-amplified using Q5 PCR enzymes (NEB) and purified with Ampure XP beads (Beckman Coulter) and purified with gel extraction. The library was sequenced using Next-Seq 500 (Illumina).The adapter sequences were removed using Cut adapt. The reads were mapped to the GRCh38 genome using STAR (36). Duplicate reads were removed using the SAM tools (38). Peak calling was performed on the processed reads using Clipper, and common peaks between the WT LIN28A and the ZFm LIN28A were extracted. The regions of peaks were analyzed with RSeQC (39), and the motifs were analyzed using Homer (40).

### Reporter assay for LIN28A-binding area

2 × 10^4^ of 293FT cells were seeded on 96-well plates. After 24 h incubation, 20 ng of luciferase reporter vector, 10 ng of Renilla vector, and 70 ng of effector vector (NC, WT, CSDm) were cotransfected with Fugene HD (Promega) and Opti-Mem (Gibco). Forty-eight hours later, the media was discarded and the luciferase activities were measured with ARVO X3 (PerkinElmer). Six replicated samples were used for the measurement of each sample (N = 6).

### *In vivo* xenograft microvessels density assay

HeLa cells (1 × 10^7^) infected with lentivirus expressing GFP or the WT ZFm of LIN28A were transplanted subcutaneously using 100 μl PBS in a 6-week-old female nude mice. The transplanted tumor was grown until it reached a size of 3 cm × 3 cm. Three independent experiment was performed for each sample (N = 3). The extracted tumors were fixed with 4% paraformaldehyde phosphate buffer solution overnight and embedded in paraffin. The embedded samples were sliced and immunostained with anti-CD34 antibody (MEC14.7) (Abcam) as the primary antibody and rabbit anti-Rat IgG (H + L), biotinylated (BA-4000) (Vector Laboratories) as secondary antibodies. Ten areas of densely concentrated microvessels (hot spots) were spotted at 40× magnification (×4 objective lens and ×10 ocular lens). In each case, these hot spots were used to count the microvessels at 200× magnification (20× objective lens, 10× ocular lens). We identified a vascular unit, a cell or group of endothelial cells of a brownish color, clearly separated from adjacent microvessels, tumor cells, and other connective tissue. Differences between negative control were evaluated by Dunnet test (N = 3).

### Statistical analysis

Statistical methods and significance values are indicated in the text and figure legends. Comparisons between groups (groups > 2) was performed using one-way ANOVA with Tukey’s test or Dunnett’s test as post hoc test.

## Data availability

The raw sequencing data were submitted to GEO under accession number No.GSE174387 (RNA-seq), GSE174388 (CLIP-seq), and DDBJ under accession number No.DRA015106 (SLAM-seq).

## Supporting information

This article contains supporting information.

## Conflict of interest

The authors declare that they have no conflicts of interest with the contents of this article.

## Figures and Tables

**Supporting Figure S5:**