# Supplementary material for: RNA-binding protein LIN28A upregulates transcription factor HIF1α by posttranscriptional regulation via direct binding to UGAU motifs
Source: J Biol Chem. 2022 Dec 9;299(1):102791. doi: 10.1016/j.jbc.2022.102791 (PMC9823215; doi:10.1016/j.jbc.2022.102791)
Supplement: Supporting Information S1 [file mmc1.docx]

**Additional experimental procedures**

**Plasmid Construction**

Plasmids expressing RNA-binding proteins used for RNA-binding protein screening were generously gifted by Naoki Goshima (41). As a negative control vector, we utilized pDESTMC-VenusA206K, the sequence of which is described in Supporting Information S2. The pLuc2-KAP-MCS vector was generated using a previously reported method (42). Using the pLuc2-KAP-MCS backbone, we created a HIF1A luciferase reporter vector by inserting the HIF1A 5’UTR before the luciferase gene and HIF1A 3’UTR after the luciferase gene. The let-7 sensor vector was created by cloning the let-7 complementary sequence between the EcoRI and XhoI sites. The luciferase reporter vector for examining motif functions was created by inserting the HBB 3’-UTR and LIN28A targeting motif sequence after the luciferase gene. The sequence encoding the ORF of human LIN28A and its zinc knuckle domain mutant was cloned with a FLAG sequence into pcDNA3.1(+) (Thermo Fisher Scientific) between the NheI and EcoRI sites. The lentivirus expressing GFP and the human LIN28A WT, ZFm, and CSDm were generated by inserting the ORF of GFP and human LIN28A or its zinc knuckle domain mutant with a FLAG sequence into the pCS2 (RIKEN, Tokyo, Japan) or pCSII with a tet-on sequence (shown in Supporting Information S2) between the NheI and EcoRI sites. The sequences are shown in Supporting Information S2.

**Cell culture**

293FT cells and HeLa cells were maintained in DMEM (Corning, New York, NY, USA) supplemented with 10% fetal bovine serum (Gibco, Carlsbad, CA, USA) and 1% penicillin-streptomycin (Wako, Osaka, Japan) at 37°C with 5% CO2.

**Reanalysis of CLIP data**

We reanalyzed the deposited data from GEO dataset GSM910957 in GSE37114. The adapter of fastq data was trimmed with Trim Galore, and the quality of fastq data was checked with fastqc (<https://www.bioinformatics.babraham.ac.uk/projects/fastqc/>). The trimmed fastq data was mapped on mm10 genome with Bowtie2 software (<http://bowtie-bio.sourceforge.net/bowtie2/index.shtml>) and the output SAM data was converted and sorted as BAM file with samtools (<http://www.htslib.org/>). The bigwig data was created with bamCoverage command of deeptools (<https://deeptools.readthedocs.io/en/develop/>).

**References**

1. Y Maruyama, A Wakamatsu, Y Kawamura, K Kimura, J Yamamoto, T Nishikawa, Y Kisu, S Sugano, N Goshima, T Isogai and N Nomura, Human Gene and Protein Database (HGPD): a novel database presenting a large quantity of experiment-based results in human proteomics, *Nucleic Acids Res*, **37**, 2009, D762-766.
2. Y Ito, A Inoue, T Seers, Y Hato, A Igarashi, T Toyama, KD Taganov, MP Boldin and H Asahara, Identification of targets of tumor suppressor microRNA-34a using a reporter library system, *Proc Natl Acad Sci U S A*, **114**, 2017, 3927–3932.
